# The Molecular Karyotype of 25 Clinical-Grade Human Embryonic Stem Cell Lines

**DOI:** 10.1038/srep17258

**Published:** 2015-11-26

**Authors:** Maurice A. Canham, Amy Van Deusen, Daniel R. Brison, Paul A. De Sousa, Janet Downie, Liani Devito, Zoe A. Hewitt, Dusko Ilic, Susan J. Kimber, Harry D. Moore, Helen Murray, Tilo Kunath

**Affiliations:** 1 MRC Centre for Regenerative Medicine, Institute for Stem Cell Research, School of Biological Sciences, The University of Edinburgh, UK; 2 Department of Reproductive Medicine, St. Mary’s Hospital, Central Manchester NHS Foundation Trust, Manchester Academic Health Sciences Centre, Manchester, UK; 3 Roslin Cells Limited, Nine Edinburgh BioQuarter, Edinburgh, UK; 4 Centre for Clinical Brain Sciences and MRC Centre for Regenerative Medicine, The University of Edinburgh, UK; 5 Stem Cell Laboratories, Guy’s Assisted Conception Unit, Division of Women’s Health, Faculty of Life Sciences and Medicine, King’s College London, London, UK; 6 Centre for Stem Cell Biology, Department of Biomedical Science, The University of Sheffield, Sheffield, UK; 7 Faculty of Life Sciences, The University of Manchester, Manchester, UK

## Abstract

The application of human embryonic stem cell (hESC) derivatives to regenerative medicine is now becoming a reality. Although the vast majority of hESC lines have been derived for research purposes only, about 50 lines have been established under Good Manufacturing Practice (GMP) conditions. Cell types differentiated from these designated lines may be used as a cell therapy to treat macular degeneration, Parkinson’s, Huntington’s, diabetes, osteoarthritis and other degenerative conditions. It is essential to know the genetic stability of the hESC lines before progressing to clinical trials. We evaluated the molecular karyotype of 25 clinical-grade hESC lines by whole-genome single nucleotide polymorphism (SNP) array analysis. A total of 15 unique copy number variations (CNVs) greater than 100 kb were detected, most of which were found to be naturally occurring in the human population and none were associated with culture adaptation. In addition, three copy-neutral loss of heterozygosity (CN-LOH) regions greater than 1 Mb were observed and all were relatively small and interstitial suggesting they did not arise in culture. The large number of available clinical-grade hESC lines with defined molecular karyotypes provides a substantial starting platform from which the development of pre-clinical and clinical trials in regenerative medicine can be realised.

Since the derivation of human embryonic stem cells (hESCs) from blastocysts in 1998^1^, and the more recent production of human induced pluripotent stem cells (iPSCs) from adult tissues^2^, anticipation has been growing with regard to their potential as cell therapies for a number of incurable conditions. As with any new medicine, Good Manufacturing Practice (GMP) is required to produce hESC/iPSC-derived cell products for clinical use in humans^3^. However, while over 1200 hESC lines have been established and reported worldwide^4^, the majority are suitable only for research purposes due to the sourcing of embryonic material, derivation process and subsequent handling procedures. Frequently, derivation and culture methods employ mouse feeder cells or poorly defined media containing animal-based products^1^,^5^, which may render these cell lines unusable as a starting material for any cell-based clinical application.

In recent years, there have been advances in the derivation of hESC lines whereby fully defined media devoid of animal-derived products is used^6^,^7^, and the traditional mouse feeders have been replaced with GMP-qualified human feeders^8^,^9^,^10^ or recombinant human proteins as a substrate on which to culture hESCs^11^,^12^,^13^,^14^. Furthermore, animal-based enzymes and guinea pig complement used to isolate the inner cell mass for hESC derivation have been replaced with mechanical isolation or laser microdissection^15^,^16^,^17^,^18^,^19^. These efforts have culminated in the derivation of approximately 50 clinical-grade hESC lines from various centres across the world^20^,^21^,^22^,^23^ (www.mrc.ac.uk/research/facilities/stem-cell-bank; stemcells.nih.gov).

Remarkably, 38 of these lines have been derived among five different centres in the United Kingdom through funding from the Medical Research Council (MRC), Scottish Enterprise, the North West Development Agency and the Juvenile Diabetes Research Foundation. The MRC launched an initiative in 2005 to provide infrastructure funding to UK *in vitro* fertilization (IVF) units to provide GMP-compliant embryos for hESC line derivation and further funded the Human Embryonic Stem Cell Co-ordinators (hESCCO) network, subsequently the National Clinical hESC Forum. This allowed the derivation centres to work with the Human Fertilisation and Embryology Authority and the UK Stem Cell Bank to establish common parameters for patient consent, screening and embryo procurement for the derivation of clinical-grade hESC lines. Ultimately this farsighted policy has yielded a cohort of hESC lines which have benefited from the shared implementation of GMP-compliant IVF laboratory standards, hESC derivation procedures and ethical principles for donor consenting^24^. A list of clinical-grade hESC lines conforming to the European Union Tissue and Cells Directives (Directives 2004/23/EC and 2006/17/EC) is shown in Table 1. These directives introduced common safety and quality standards across European member states to ensure that all tissues and cells used in patient treatment are traceable from donor to recipient, thus implementing key principles of GMP.

The value of a large number of different cell lines as starting material for clinical applications is three-fold: (a) different hESC lines have varying propensities to generate specific cell lineages during *in vitro* differentiation^25^, (b) hESC lines may harbour or acquire genetic anomalies potentially excluding them from clinical use^26^, and (c) in order to accommodate human leucocyte antigen (HLA) matching to a broad section of potential patients a sizeable number of hESC lines would be required. It has been estimated that approximately 150 different lines with particular HLA haplotypes would be required to cover ~93% of potential UK recipients^27^. With the advent of iPSC technology, the latter issue will be addressed by derivation of hiPSC lines from individuals homozygous for common HLA loci. An international effort is currently underway to address this^28^, but the issues of line-to-line variation and genetic stability of hESC and iPSC lines will remain^29^.

In accordance with GMP standards applicable to the sourcing and the application of raw materials used during production processes, such as hESC/iPSC cell derivation and differentiation into desired cell types, there is emerging evidence that equal scrutiny should be undertaken to account for the genetic health of cells at all stages of production^3^,^30^. Some characteristics of self-renewal and multipotency that define pluripotent stem cells (PSCs) are also shared by tumour stem cells^31^. This has been exemplified by the identification of non-random genetic changes, particularly gains of chromosomes 12, 17 and X, common to embryonal carcinoma cell lines and PSCs following prolonged culture^26^,^32^. Moreover, recent studies using high-resolution single nucleotide polymorphism (SNP) analysis of PSCs have shown copy number variations (CNVs) that can lead to gene expression alterations functionally linked to cancer^33^. One such microduplication associated with oncogenic transformation was detected on chromosome 20q11.21^34^,^35^. A comprehensive survey of 125 different PSC lines from many different laboratories across the world showed that about one third of lines acquired a culture-induced genomic variation upon prolonged culture, the most common of which was chromosome 20q11.21 microduplication^36^. The anti-apoptotic gene, *BCL2L1*, within this region has been shown to be a driver of growth advantage^36^,^37^. In addition to anomalies acquired during self-renewal, the process of *in vitro* differentiation from genetically healthy PSCs can also lead to genomic instability^38^.

However, most CNVs are benign and relatively large duplications and deletions (>100 kb) are common in healthy individuals^39^. Such parent-of-origin CNVs will also be present in blastocysts and hESC lines derived from them. The ideal way to determine if a CNV identified in a hESC line is naturally occurring is to genotype the parents of the donated blastocyst. This is indeed possible in cases involving preimplantation genetic diagnosis^40^. However, the vast majority of blastocysts, including all within the UK, are donated under conditions that prohibit access to parental DNA. An alternative method to determine if a particular CNV observed in a hESC cell line might be parent-of-origin is to compare it to known CNVs present on the Database of Genomic Variants (DGV)^41^. The DGV has catalogued a collection of published CNVs from over 14,300 healthy individuals. Although not exhaustive, the collection is highly curated and covers a significant number of CNVs and other genomic structural variants known to exist in the general population. However, if a hESC CNV is found to be present on this database it does not exclude the possibility that it is a *de novo* genomic alteration that arose during development of the blastocyst or during establishment and maintenance of the cell line.

As well as CNVs, copy-neutral loss of heterozygosity (CN-LOH) represent another form of genomic structural variation characterised by a stretch of homozygosity along part of a chromosome^42^. If the affected alleles contain recessive mutations or lie within regions of the genome subject to imprinting, there can be either a net loss or a net gain of gene function and expression^42^. CN-LOH regions can also be due to the presence of persistent ancestral recombination ‘cold spots’ or be the consequence of recent consanguinity^43^,^44^. While these changes would be considered parent-of-origin if found in hESC lines, there are examples of somatic or acquired CN-LOH regions found during the progression of many cancers, particularly those of haematopoietic origin^45^. Whole genome SNP arrays are useful to detect CN-LOH events instead of regions, and regions greater than 1 Mb in length warrant further investigation^42^.

In this study, we sought to examine the genetic integrity of 25 clinical-grade hESC lines utilising whole-genome SNP genotyping analysis. While karyology is sufficient for establishing genetic normalcy within current regulatory standards, advances in technology and an increasing cytogenetic knowledge base demand higher resolution investigation of cell lines and cell products designed for clinical use.

## Results and Discussion

**Figure 1:**
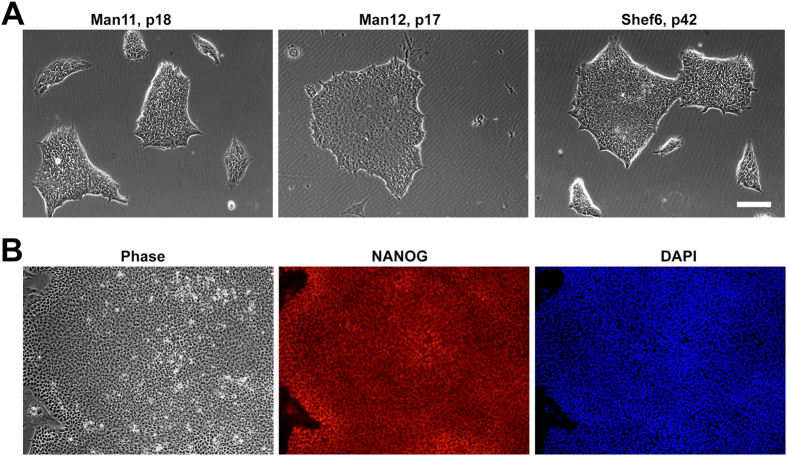
Culture of 3 clinical-grade hESC lines. (**A**) Man11, Man12, and Shef6 hESCs were cultured in Laminin-521 in Essential 8 medium prior to collection of genomic DNA. (**B**) Human ESC lines maintained expression of the pluripotent marker, NANOG, during expansion. Scale bar, 90 μm.

Human ES cell lines were cultured in feeder-free conditions prior to the isolation of genomic DNA (Fig. 1). The DNA from 25 hESC lines (Table 1) was assayed for single nucleotide polymorphisms (SNPs) using the Illumina HumanCytoSNP-12 array and data was analysed for large CNVs with GenomeStudio and KaryoStudio software. Our SNP analysis of 25 clinical-grade hESC lines found 15 unique CNVs greater than 100 kb and 3 CN-LOH regions greater than 1 Mb in size among 16 hESC lines, with results summarised in Table 2. Nine clinical-grade hESC lines did not harbour any structural genomic variants of this size. The percentage of cell lines we found to have CNVs greater than 100 kb (72%) is in agreement with the percentage of healthy individuals (~70%) in the population found to harbour CNVs of at least this size^39^. Additionally, the percentage of cell lines with CN-LOH events between 2.5 and 5 Mb (12%) is similar to the percentage of individuals in outbred populations with CN-LOH of this size range^43^. Since we have restricted our search to large structural changes, we are reporting a considerably lower number of CNVs and CN-LOH regions identified in other studies that have examined hESC or hiPSC lines^33^,^36^,^46^. Approximately 5%–10% of the normal human genome contains CNVs averaging a few kilobases in length^47^, and high resolution arrays can produce large data sets dominated by such naturally occurring small events^48^,^49^. Thus, we chose to use the HumanCytoSNP-12 array and KaryoStudio software, tailored to identify CNVs greater than 75 kb and CN-LOH regions greater than 1 MB, a resolution adequate for common molecular cytogenetic interpretation and applicable in a clinically relevant setting^42^,^49^,^50^,^51^.

Of the 15 unique CNVs detected, 12 were heterozygous duplications, 2 were heterozygous deletions, and 1 was a homozygous deletion (Table 2). We asked whether these structural genomic variants were likely to be parent-of-origin CNVs, that is, naturally occurring, or if they could have arisen during the hESC derivation process or during expansion in culture. We first checked each CNV on the DGV (http://dgv.tcag.ca) to determine whether the CNVs have been previously observed in healthy individuals^41^,^52^.

**Figure 2:**
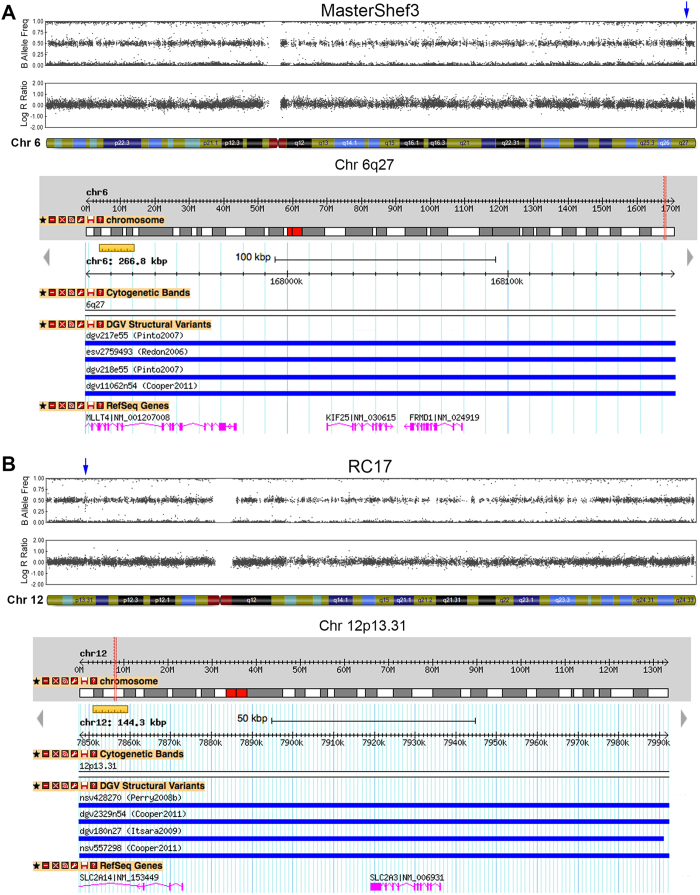
Duplications found in hESC lines that are present on the DGV. (**A**) Chromosome 6 ideograms from SNP array analysis of MasterShef3 revealed a 267 kb duplication near the telomere, which contained 3 genes, *MLLT4, KIF25,* and *FRMD1*. Duplications of this size, or greater, have been reported and annotated on the DGV with an estimated frequency of 2.82% in the human population. (**B**) A 144 kb duplication was observed on chromosome 12p13.31 of RC17 hESCs. This region contained two genes, *SCL2A14* and *SLC2A3*, and is represented on the DGV (3.9% frequency in humans).

Amongst the 15 large hESC CNVs, we found 10 had clear evidence of being present in healthy individuals. For example, a duplication of 267 kb on chromosome 6q27 observed in MasterShef3 containing 3 protein-encoding genes—*MLLT4, KIF25, FRMD1*—was represented on the DGV and has been reported in the healthy population at a frequency of over 1 in 50 individuals (Fig. 2A) 48,51,53. RC17 hESCs harboured a single 144 kb duplication on chromosome 12p13.31 encompassing the *SLC2A14* and *SLC2A3* genes (Fig. 2B). Although this is close to the *NANOG* locus, we do not believe it confers a growth advantage since this CNV is commonly found (1 in 25) in healthy individuals^39^,^51^,^54^. A 132 kb duplication on chromosome 12p11.21 was detected in both KCL033 and KCL040 hESC lines (Supplementary Fig. S1). This region does not contain any protein-coding genes, and there are at least 14 submissions of this duplication on the DGV^39^,^51^,^53^,^54^,^55^. Man11, MasterShef2, and MasterShef7 also harboured genomic duplications of greater than 100 kb that are represented on the DGV (Supplementary Fig. S1). Man11 harboured a 220 kb gain on chromosome 15q25.3 that has been reported several times^39^,^51^,^56^. This duplication contains one gene, *AKAP13,* and this CNV was not found in the sibling hESC line, Man12. One of the CNVs detected in MasterShef7, a 315 kb duplication present on chromosome 14q21.3, contains a single gene, *MDGA,* and this CNV is also present on the DGV^51^. A 572 kb gain on chromosome 17q21.31 encompassing 5 genes in MasterShef2 was also found to be present in the normal population at high frequency (9.8%)^53^.

**Figure 3:**
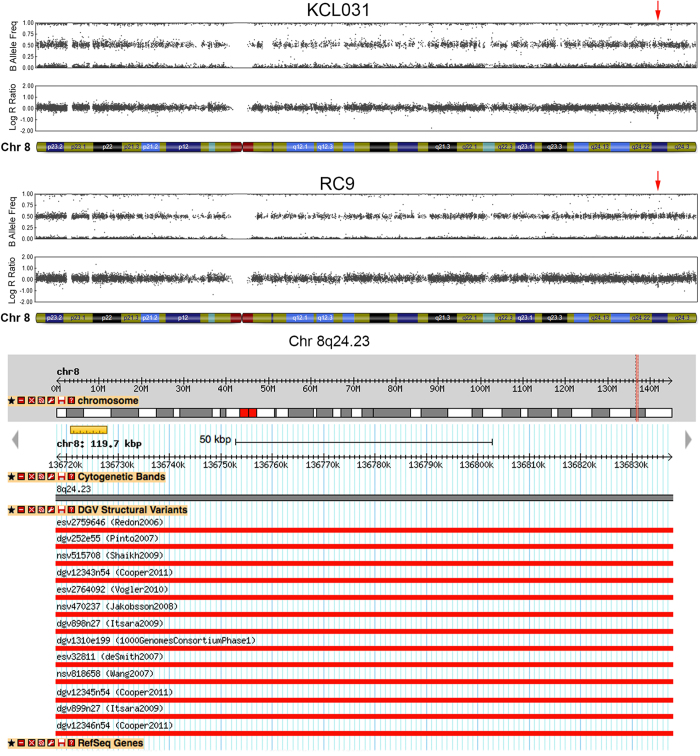
A common deletion observed in two unrelated hESC lines. Chromosome 8 ideograms from SNP array analysis of KCL031 and RC9 hESC lines revealed a 120 kb deletion on chromosome 8q24.23 (red arrow). This deletion is relatively common in the human population (3.85%), and does not contain any protein-coding genes.

Both heterozygous deletions and the homozygous deletion were found to be naturally occurring in the human population. Two unrelated cell lines, KCL031 and RC9 hESCs harboured the same 120 kb deletion on chromosome 8q24.23 (Fig. 3). This CNV is estimated to have a frequency of about 1 in 26 people and has been reported numerous times to occur in healthy individual^39^,^48^,^51^,^53^,^55^,^56^. This region does not contain any protein-coding genes. KCL040 and MasterShef11 also possessed genomic deletions greater than 100 kb that were present on the DGV (Supplementary Fig. S2). KCL040 harbours a previously reported 1.5 Mb deletion on chromosome 16p11.2 that contains 3 related genes^56^,^57^. MasterShef11 has a 109 kb homozygous deletion on chromosome 19p12 that has been widely reported to occur in healthy individuals (~1 in 9) and does not contain any genes^39^,^48^,^51^,^55^,^56^.

**Figure 4:**
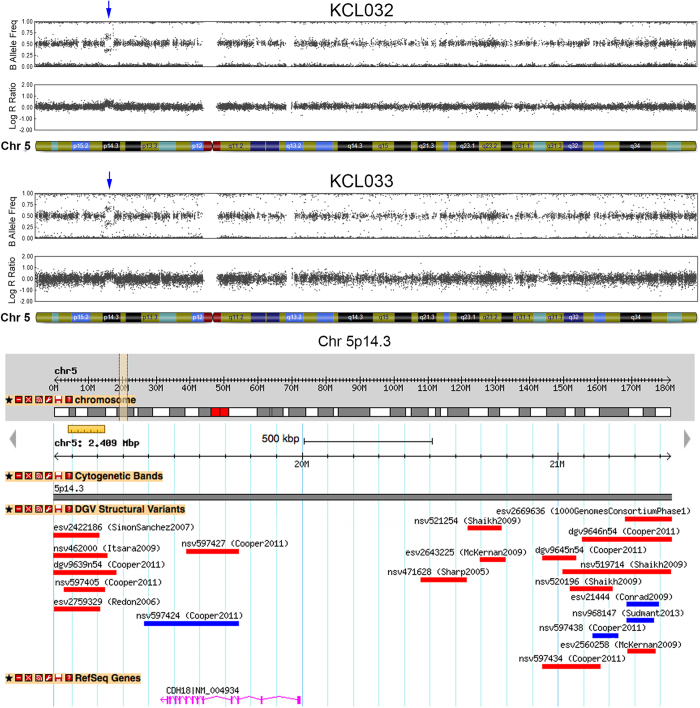
An identical duplication found in sibling KCL hESC lines, but not present on the DGV. Chromosome 5 ideograms from SNP array analysis of KCL032 and KCL033 hESC lines with the common CNV indicated by blue arrows. This 2.4 Mb duplication on chromosome 5p14.3 included the *CDH18* gene. A duplication of this size was not present on the DGV, but a smaller duplication (nsv597424) covering most of the *CDH18* exons was present, and a number of smaller deletions have been observed in this region. Only published CNVs greater than 100 kb are represented here.

We identified a novel 2.4 Mb gain on chromosome 5p14.3, containing a single gene, *Cadherin-18*, that was present in two sibling cell lines, KCL032 and KCL033 (Fig. 4), but not in KCL034, a third sibling line^20^,^58^. A duplication of this size has not been reported to date, but its presence in two sibling hESC lines strongly suggests it was inherited from one of the parents of the donated blastocysts rather than by acquisition of an identical CNV during hESC derivation and culture.

**Figure 5:**
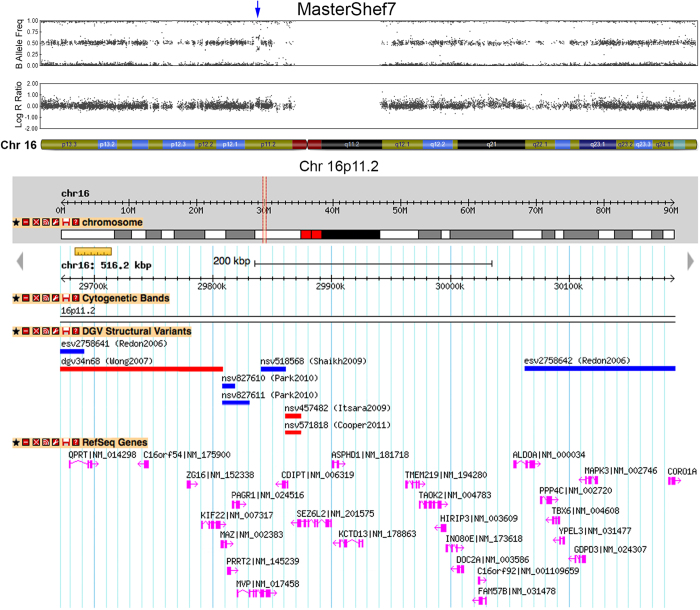
A novel duplication observed in MasterShef7. A 516 kb duplication on chromosome 16p11.2 was detected in MasterShef7 by SNP array analysis. This region contained 26 protein-coding genes, and a duplication of this size has not been reported to date. Smaller duplications have been observed in this region, including a duplication (esv2758642) spanning 7 of the genes. Only published CNVs greater than 10 kb are represented here.

The remaining 5 CNVs, all duplications, were not fully represented on the DGV. For example, a 516 kb duplication was detected on chromosome 16 in MasterShef7 hESCs that encompassed over 20 genes (Fig. 5). A similarly sized duplication of this region has not been reported to date, but the DGV is not exhaustive and this CNV may represent a novel, but rare naturally-occurring genomic variant. This duplication is not known to confer a selective growth advantage, and has not been reported to be associated with hESC culture adaptation^36^. We also checked this CNV on the DECIPHER database^59^ of microdeletion and microduplication clinical syndromes to determine if it was associated with a known disorder. This CNV was not associated with a described clinical syndrome, nor were any of the other 14 CNVs identified here.

The four other unique CNVs that were not fully represented on the DGV were present in KCL034, KCL037, MasterShef3, and Shef6 hESC lines (Supplementary Fig. S3). KCL034 harboured a duplication of a 331 kb region on chromosome 6 (chr6q22.1) that contained part of the Histone 1 gene cluster. While this region was not fully present on the DGV, it is probable that this gain represents a benign event as other histone clusters have been shown to be preferentially duplicated during evolution^60^. A 542 kb gain on chromosome 18q23 in KCL037 containing two coding genes, *SALL3* and *ATP9B*, has not been previously reported. However, a smaller duplication covering the same two genes has been observed^51^. A 235 kb duplication was detected on chromosome 17 in MasterShef3 hESCs. Although, a duplication of this size is not present on the DGV, four slightly smaller duplications of the region have been reported^39^,^51^,^53^,^55^. A 553 kb gain on chromosome 8p22 in Shef6 hESCs within an intron of the *SGCZ* gene is an unreported novel structural duplication, but a deletion of this region has been observed^51^. While the 5 novel CNVs detected in our study were not fully present on the DGV, they were also not on the ‘ESC-associated’ culture-adaptation list of CNVs from the International Stem Cell Initiative survey of 125 different hESC and hiPSC lines^36^. Based on the available evidence, these CNVs likely represent novel, but rare, structural variants found in the human population. However, we do not know the health status of the individuals that may harbour these novel CNVs, so we cannot assume they are benign. Furthermore, we cannot definitively exclude the possibility that these CNVs arose during blastocyst development or during the early stages of hESC line derivation.

**Figure 6:**
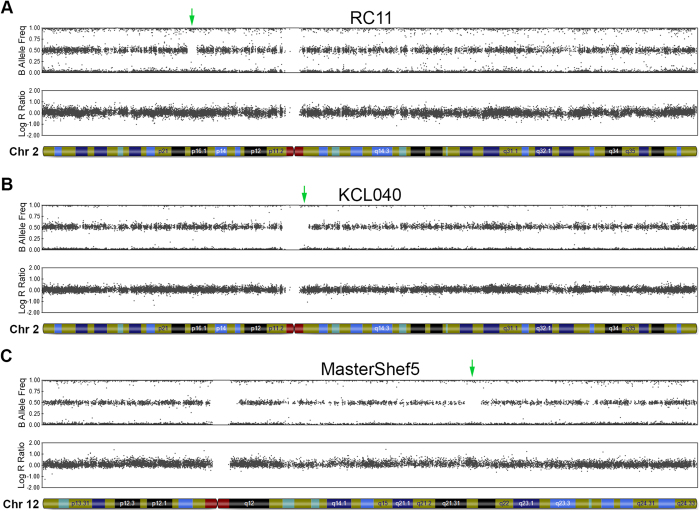
Interstitial CN-LOH regions detected in clinical-grade hESC lines. Chromosome ideograms from SNP array analysis showing CN-LOH regions indicated by green arrows: (**A**) A 3.6 Mb region on chromosome 2p16.2-16.1 in RC11. (**B**) A 3.5 Mb region on chromosome 2q11.1-11.2 in KCL040. (**C**) A 3.4 Mb region around chromosome 12q21.31-21.33 in MasterShef5.

In addition to the 15 CNVs identified, our analysis detected 3 regions of CN-LOH greater than 1 MB among three different hESC lines, KCL040, MasterShef5, and RC11 (Table 2 and Fig. 6). All three regions ranged in size between 3.4 and 3.7 Mb, two of which were on chromosome 2 and the other on chromosome 12. Due to their interstitial nature and relatively small genetic size, it is unlikely any of these CN-LOH regions would represent examples of acquired CN-LOH. The vast majority of CN-LOH events documented in cancers are telomeric in nature and while examples of acquired interstitial CN-LOH do exist^61^,^62^, the double recombination event required to achieve this would be difficult to explain for sizes less than 25 Mb^63^,^64^. Since hESCs are known to maintain monoallelic expression in some imprinted regions^65^, we cross-referenced the 3 CN-LOH regions we identified to the Genomic Imprinting database (http://www.geneimprint.com)^66^. None of the CN-LOH regions reside in known imprinted regions, although *CCDC85A* in the CN-LOH of RC11 is predicted, but not validated, to be an imprinted gene on this database.

While none of the CNV and CN-LOH regions observed in the hESCs appear to harbour genomic anomalies associated with culture adaptation at the passages reported here (Table 2), we have detected the presence of the culture-adapted microduplication on chromosome 20q11.21 at higher passages of 4 clinical-grade hESC lines. This microduplication was reported to be found in ~20% of research-grade hESC lines^36^,^37^. Thus, it is with prudence that each cell line should be re-evaluated frequently and certainly before the production of any differentiated cell product^67^. These results also illustrate that the heterogeneity of molecular karyotypes in the human population will be reflected in the cell lines produced from human embryos. A perfect genome is unlikely to exist, so an appreciation of human genomic diversity will lend itself to a more measured interpretation of molecular karyotype and genome sequencing data of cell lines destined for clinical use.

Our molecular karyotypic evaluation of 25 clinical-grade hESC lines has established a valuable platform for the development and manufacture of cell therapy products for clinical application in regenerative medicine.

## Materials and Methods

### Clinical-grade hESC Samples

Approval for the use of all hESC lines used in this study was granted by the MRC Steering Committee for the UK Stem Cell Bank and for the Use of Stem Cell Lines. The following clinical-grade hESC lines were kindly provided by the following derivation centres: The University of Sheffield (12 lines) MasterShef2, MasterShef3, MasterShef4, MasterShef5, MasterShef7, MasterShef8, MasterShef10, MasterShef11, MasterShef12, MasterShef13, MasterShef14 and Shef6; King’s College London (8 lines) KCL031, KCL032, KCL033 KCL034, KCL037, KCL038, KCL039, and KCL040; Roslin Cells Ltd (3 lines) RC9, RC11, and RC17; and Central Manchester NHS Foundation Trust/The University of Manchester (2 lines) Man11 and Man12. Most of the cell samples were provided as frozen cell pellets, which were directly processed for genomic DNA isolation. However, Man11 and Man12, sibling hESC lines, were provided as cryopreserved hESC lines, and Shef6 was obtained directly from the UK Stem Cell Bank (UKSCB Accession No. R-05-031). These three lines were thawed and cultured in Essential 8 media (Life Technologies) on Laminin-521 substrate (Biolamina) for less than 10 passages before pelleting by centrifugation for genomic DNA isolation. Human ES cell morphology and pluripotent marker, NANOG, expression were maintained during this expansion (Fig. 1). NANOG immunostaining was performed with anti-NANOG antibody (1:500) from R&D Systems (cat no. AF1997).

### Isolation of Genomic DNA

Genomic DNA was isolated from cell pellets using the MasterPure™ Complete DNA and RNA Purification Kit (Epicentre) according to the manufacturer’s instructions. Briefly, cell pellets were lysed with Tissue and Cell Lysis Solution, followed by Proteinase K and RNase A treatment. Proteins were precipitated with MPC Protein Precipitation Reagent and removed by centrifugation. Genomic DNA was precipitated with isopropanol, pelleted by centrifugation and then resuspended in TE buffer to a final concentration of 50 ng/μl. Purity was checked by spectrophotometry using the NanoDrop 1000 spectrophotometer (Thermo Fisher Scientific).

### HLA typing

All samples were subjected to HLA typing at the Histocompatibility and Immunogenetics (H&I) Laboratory of the Scottish National Blood Transfusion Service (SNBTS). The HLA typing data (Supplementary Table S1) was used for identification purposes by comparing it to the HLA typing data for each cell line provided by the hESC derivation centres. The H&I Laboratory at SNBTS is accredited through Clinical Pathology Accreditation (UK) Ltd (CPA), and all CPA labs are subjected to UK National External Quality Assessment Schemes (UK NEQAS).

### Genotyping and Analysis

Genomic DNA samples were assayed using the Illumina HumanCytoSNP-12 v2.1 BeadChip, at either AROS (Aarhus, Denmark) or the Wellcome Trust Clinical Research Facility (Edinburgh, UK). The data have been deposited in NCBI’s Gene Expression Omnibus and are accessible through GEO Series accession number GSE68508. Genotyping was initially assessed using GenomeStudio genotyping module (v1.94, Illumina). KaryoStudio (v1.4, Illumina) was employed to perform automatic normalisation and to identify genomic aberrations utilising default settings of the built-in cnvPartition algorithm (3.07, Illumina) to generate B-allele frequency and smoothened Log R ratio plots for detected regions. These parameters are designed to detect CNVs greater than 75 kb and CN-LOH regions larger than 1 MB with a confidence value greater than 35. All identified duplication and deletions were first cross-matched to the Database of Genomic Variants (DGV; http://dgv.tcag.ca) to identify naturally-occurring structural variations in the human genome^41^,^52^. We also determined the estimated frequency of 8 common CNVs in the human population (Supplementary Table S2) by accessing the DGV Gold Standard track of a highly curated and accurate CNV map of the human genome^47^. All CNVs were inputted into the DECIPHER database (https://decipher.sanger.ac.uk/) to determine if they were associated with any clinical syndromes^59^. The CN-LOH regions were cross-referenced with the Genomic Imprinting database (http://www.geneimprint.com) to determine if the genomic variants occurred in known imprinted regions^66^. CNVs that were not identified on the DGV were then checked against a list of ES cell-associated culture adaptation genomic variants published by the International Stem Cell Initiative^36^.

## Additional Information

**How to cite this article**: Canham, M. A. *et al.* The Molecular Karyotype of 25 Clinical-Grade Human Embryonic Stem Cell Lines. *Sci. Rep.* **5**, 17258; doi: 10.1038/srep17258 (2015).

## Supplementary Material

Supplementary Information

## Figures and Tables

**Table 1:** List of 38 EUTCD compliant (clinical-grade) hESC lines.

Derivation Centre	EUTCD Grade Cell Lines	Number
King’s College London^1^	**KCL031**, **KCL032**, **KCL033**, **KCL034**, **KCL037**, **KCL038**, **KCL039**, **KCL040**	8
The University of Manchester	**Man11**, **Man12**, *Man13*, *Man14*, *Man15*, *Man16*	6
Newcastle University	*NCL14*	1
Roslin Cells Ltd, Edinburgh	**RC9**, **RC11**, *RC12*, *RC13*, *RC14*, *RC15*, *RC16*, **RC17**	8
The University of Sheffield	*MasterShef1*, **MasterShef2**, **MasterShef3**, **MasterShef4**, **MasterShef5**, *MasterShef6*, **MasterShef7**, **MasterShef8**, **MasterShef10**, **MasterShef11**, **MasterShef12**, **MasterShef13**, **MasterShef14**, *Shef3.2,* **Shef6.1**	15
Total		**38**

The 25 hESC lines analysed by SNP analysis are shown in bold.

^1^All eight (8) of the KCL hESC lines are listed on the NIH Stem Cell Registry (escr.nih.gov) making them available for NIH-funded projects in the USA.

**Table 2:** Summary of SNP array analysis of clinical-grade hESC lines.

hESC Line	Passage	Detected Region	Variation	Start (bp)^1^	End (bp)	Length (kb)	# SNPs	*Genes*
KCL031	21	8q24.23	Loss^2^	136718037	136837768	119.7	20	NONE
KCL032	7	5p14.3	Gain	19086546	21585311	2498.8	252	*CDH18*
KCL033	17	5p14.3	Gain	19031726	21440254	2408.5	256	*CDH18*
KCL033	17	12p11.21	Gain^2^	31116366	31248444	132.1	19	NONE
KCL034	19	6p22.1	Gain	27627265	27958049	330.8	13	*HIST1H2BL; HIST1H2AI; HIST1H3H; HIST1H2AJ; HIST1H2BM; HIST1H4J; HIST1H4K; HIST1H2AK; HIST1H2BN; HIST1H2AL; HIST1H1B; HIST1H3I; HIST1H4L; HIST1H3J; HIST1H2AM; HIST1H2BO; OR2B2; OR2B6*
KCL037	8	18q23	Gain	78611768	79153453	541.7	84	*SALL3; ATP9B*
KCL040	21	2q11.1-11.2	CN-LOH	94871756	98412364	3540.6	345	*TEKT4; MAL; MRPS5; ZNF514; ZNF2; PROM2; KCNIP3; FAHD2A; TRIM43; ANKRD36C; GPAT2; ADRA2B; ASTL; DUSP2; STARD7; TMEM127; CIAO1; SNRNP200; ITPRIPL1; NCAPH; NEURL3; ARID5A; KANSL3; FER1L5; LMAN2L; CNNM4; CNNM3; ANKRD23; ANKRD39; SEMA4C; FAM178B; FAHD2B; ANKRD36; ANKRD36B; COX5B; ACTR1B; ZAP70; TMEM131; VWA3B; CNGA3*
KCL040	21	12p11.21	Gain^2^	31116366	31248444	132.1	19	NONE
KCL040	21	16p11.2	Loss^2^	32491547	33993220	1501.7	34	*TP53TG3; TP53TG3C; TP53TG3B*
Man11	21	15q25.3	Gain^2^	85376921	85597560	220.6	39	*AKAP13*
MasterShef2	18	17q21.31	Gain^2^	46138530	46710944	572.4	22	*KANSL1; LRRC37A; ARL17B; LRRC37A2; ARL17A; NSF*
MasterShef3	22	6q27	Gain^2^	167908790	168175598	266.8	59	*MLLT4; KIF25; FRMD1*
MasterShef3	22	17p11.2	Gain	21358248	21593333	235.1	31	*KCNJ12; C17orf51*
MasterShef5	37	12q21.31-21.33	CN-LOH	85929477	89347087	3417.6	397	*MGAT4C; C12orf50; C12orf29; CEP290; TMTC3; KITLG*
MasterShef7	16	14q21.3	Gain	46731743	47047175	315.4	18	*MDGA2*
MasterShef7	16	16p11.2	Gain	29672266	30188484	516.2	94	*QPRT; C16orf54; ZG16; KIF22; MAZ; PRRT2; PAGR1; MVP; CDIPT; SEZ6L2; ASPHD1; KCTD13; TMEM219; TAOK2; HIRIP3; INO80E; DOC2A; C16orf92; FAM57B; ALDOA; PPP4C; TBX6; YPEL3; GDPD3; MAPK3; CORO1A*
MasterShef11	17	19p12	Loss^2^,^3^	20423851	20532555	108.7	9	NONE
Shef6	45	8p22	Gain	14634612	15188066	553.5	93	*SGCZ*
RC9	17	8q24.23	Loss^2^	136718037	136837768	119.7	20	NONE
RC11	18	2p16.2-16.1	CN-LOH	53624707	57243565	3618.9	499	*GPR75-ASB3; CHAC2; ERLEC1; GPR75; PSME4; ACYP2; TSPYL6; C2orf73; SPTBN1; EML6; RTN4; CLHC1; RPS27A; MTIF2; CCDC88A; CFAP36; SMEK2; PNPT1; EFEMP1; CCDC85A*
RC17	17	12p13.31	Gain^2^	7847740	7992065	144.3	20	*SLC2A14; SLC2A3*
KCL038	9	NONE						
KCL039	8	NONE						
Man12	20	NONE						
MasterShef4	35	NONE						
MasterShef8	19	NONE						
MasterShef10	22	NONE						
MasterShef12	16	NONE						
MasterShef13	11	NONE						
MasterShef14	13	NONE						

^1^Nucleotide numbers refer to Human Genome Build 38.

^2^denotes that the frequency of this CNV in the human population has been estimated (Supplementary Table S2).

^3^denotes a homozygous deletion.
