# Supplementary material for: The Molecular Karyotype of 25 Clinical-Grade Human Embryonic Stem Cell Lines
Source: Sci Rep. 2015 Nov 26;5:17258. doi: 10.1038/srep17258 (PMC4660465; doi:10.1038/srep17258)
Supplement: Supplementary Information [file srep17258-s1.doc]

**SUPPLEMENTARY INFORMATION**

**The Molecular Karyotype of 25 Clinical-Grade Human Embryonic Stem Cell Lines**

Maurice A. Canham, Amy Van Deusen, Daniel R. Brison, Paul De Sousa, Janet Downie, Liani Devito, Zoe A. Hewitt, Dusko Ilic, Susan J. Kimber, Harry D. Moore, Helen Murray, Tilo Kunath

**Supplementary Table S1**

| **Cell Line** | **HLA-I** | | | | **HLA-II** | | | | |
| --- | --- | --- | --- | --- | --- | --- | --- | --- | --- |
|  | **A** | **B** | **Bw** | **C** | **DRB1** | **DRB3** | **DRB4** | **DRB5** | **DQB1** |
| KCL031 | 02, 24 | 51, 52 | 4 | 12, 14 | 11, 15 | 02 |  | 01 | 03, 06 |
| KCL032 | 02, 11 | 15, 51 | 4, 6 | 03, 04 | 04, 13 | 03 | 01 |  | 03, 06 |
| KCL033 | 11, 29 | 44, 51 | 4 | 04, 16 | 04, 07 |  | 01 |  | 02, 03 |
| KCL034 | 11, 29 | 44, 51 | 4 | 04, 16 | 04, 07 |  | 01 |  | 02, 03 |
| KCL037 | 02, 03 | 35, 40 | 6 | 03, 04 | 01, 13 | 03 |  |  | 05, 06 |
| KCL038 | 03, 11 | 07, 15 | 6 | 03, 07 | 14, 15 | 02 |  | 01 | 05, 06 |
| KCL039 | 01, 24 | 35, 49 | 4, 6 | 04, 07 | 03 | 01/02 |  |  | 02 |
| KCL040 | 03, 24 | 07, 15 | 6 | 03, 07 | 04, 15 |  | 01 | 01 | 03, 06 |
| Man11 | 02, 24 | 35, 44 | 4, 6 | 04, 05 | 04, 11 | 02 | 01 |  | 03 |
| Man12 | 01, 02 | 37, 44 | 4 | 05, 06 | 04 |  | 01 |  | 03 |
| MasterShef2 | 01, 68 | 44 | 4 | 07 | 01, 11 | 01/02/03 |  |  | 03, 05 |
| MasterShef3 | 02, 24 | 40, 44 | 4, 6 | 03, 05 | 04, 15 |  | 01 | 01 | 03, 06 |
| MasterShef4 | 01, 08 | 44 | 4, 6 | 05, 07 | 03, 15 | 01 |  | 01 | 02, 06 |
| MasterShef5 | 03, 24 | 27 | 4 | 02 | 03, 11 | 02 |  |  | 03, 02 |
| MasterShef7 | 01, 29 | 08, 44 | 4, 6 | 07, 16 | 03, 07 | 01 | 01 |  | 02 |
| MasterShef8 | 03, 23 | 14, 44 | 4, 6 | 04, 08 | 07, 13 | 03 | 01 |  | 02, 06 |
| MasterShef10 | 01, 03 | 07, 27 | 4, 6 | 01, 07 | 04, 15 |  | 01 | 01 | 03, 06 |
| MasterShef11 | 02, 03 | 07 | 6 | 07 | 15 |  |  | 01 | 06 |
| MasterShef12 | 01, 03 | 38, 40 | 4, 6 | 02, 12 | 03, 13 | 01, 02 |  |  | 02, 06 |
| MasterShef13 | 02, 11 | 18, 44 | 4, 6 | 05, 12 | 11, 15 | 02 |  | 01 | 03, 06 |
| MasterShef14 | 03, 26 | 07, 38 | 4, 6 | 07, 12 | 09, 13 | 01 | 01 |  | 03, 06 |
| Shef6 | 02, 23 | 15, 44 | 4, 6 | 03, 04 | 04, 07 |  | 01 |  | 02, 03 |
| RC9 | 01, 02 | 07, 08 | 6 | 07 | 01, 15 |  |  | 01 | 05, 06 |
| RC11 | 01, 24 | 07, 08 | 6 | 07 | 03, 15 | 01 |  | 01 | 02, 06 |
| RC17 | 01, 03 | 07, 08 | 6 | 07 | 03, 11 | 01, 02 |  |  | 02, 03 |

**Supplementary Table S1: HLA typing of 25 clinical-grade hESC lines.** Two-digit HLA typing was performed on genomic DNA isolated from 25 hESC lines.

**Supplementary Table S2**

| **Human ESC Line(s)** | **Region** | **Variation** | **GSSV No.** | **NR_freq** |
| --- | --- | --- | --- | --- |
| KCL031 and RC9 | 8q24.23 | Loss | gssv179004 | 3.85% |
| KCL033 and KCL040 | 12p11.21 | Gain | gssv32361 | 4.70% |
| KCL040 | 16p11.2 | Loss | gssv62559 | 5.14% |
| Man11 | 15q25.3 | Gain | gssv58152 | 0.30% |
| MasterShef2 | 17q21.31 | Gain | gssv71248 | 9.82% |
| MasterShef3 | 6q27 | Gain | gssv157263 | 2.82% |
| MasterShef11 | 19p12 | Loss | gssv81932 | 10.94% |
| RC17 | 12p13.31 | Gain | gssv32355 | 3.90% |

**Supplementary Table S2: Common CNVs in the human population.** The accession number of the DGV Gold Standard CNV (gssv no.) and the estimated frequency (NR_freq) in the human population are listed.

**Supplementary Figure S1A**


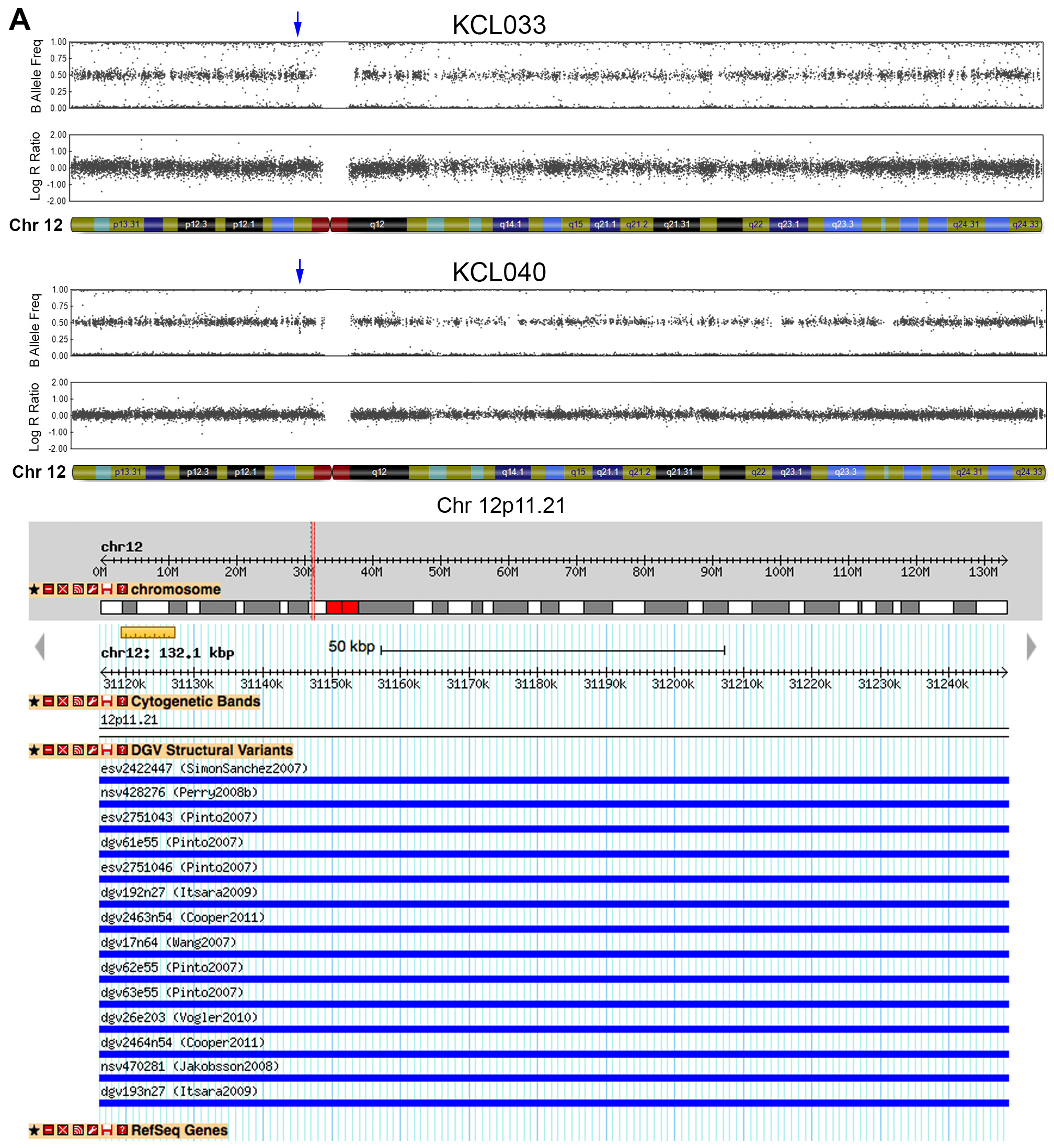


**Supplementary Figure S1B,C**


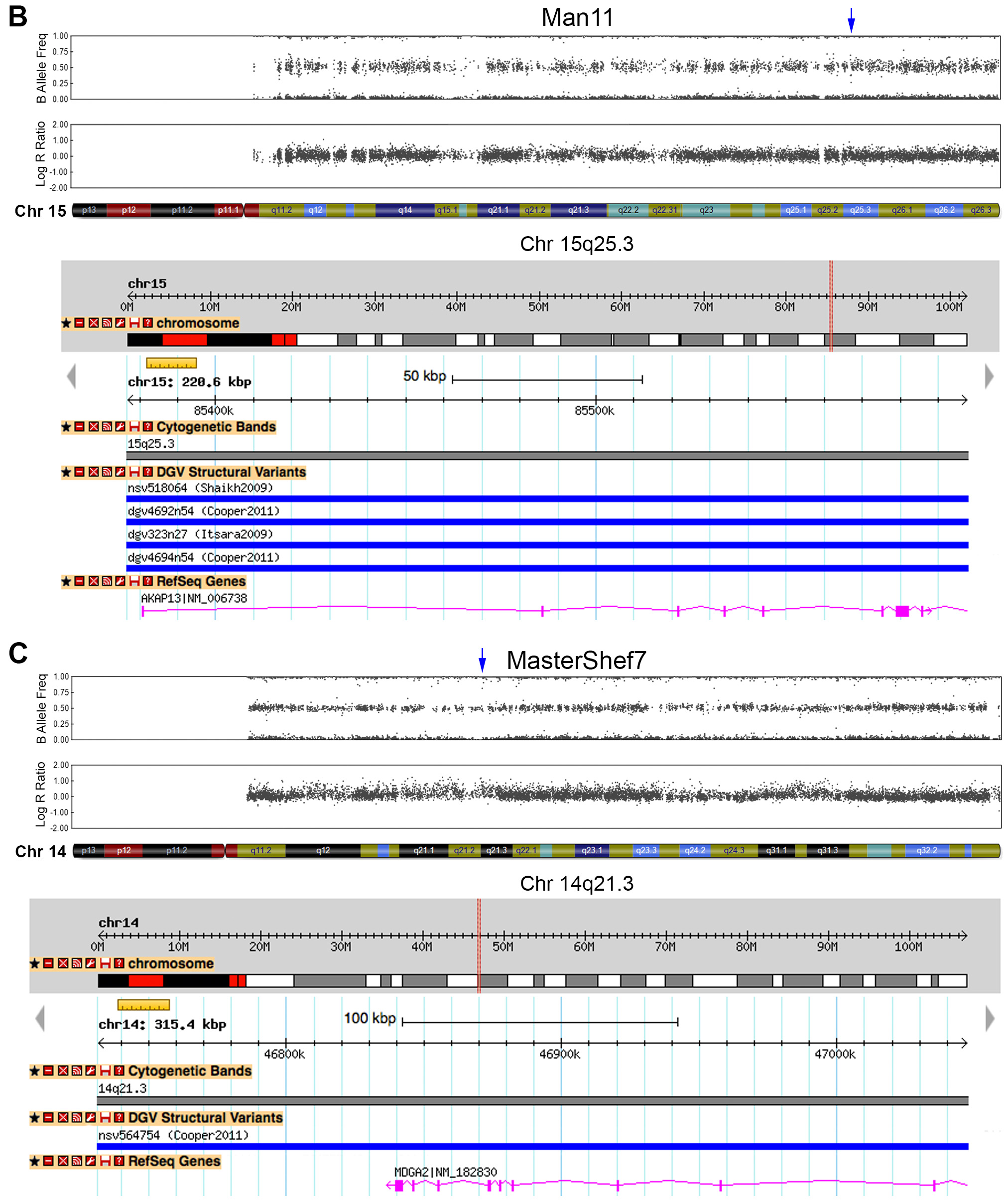


**Supplementary Figure S1D**


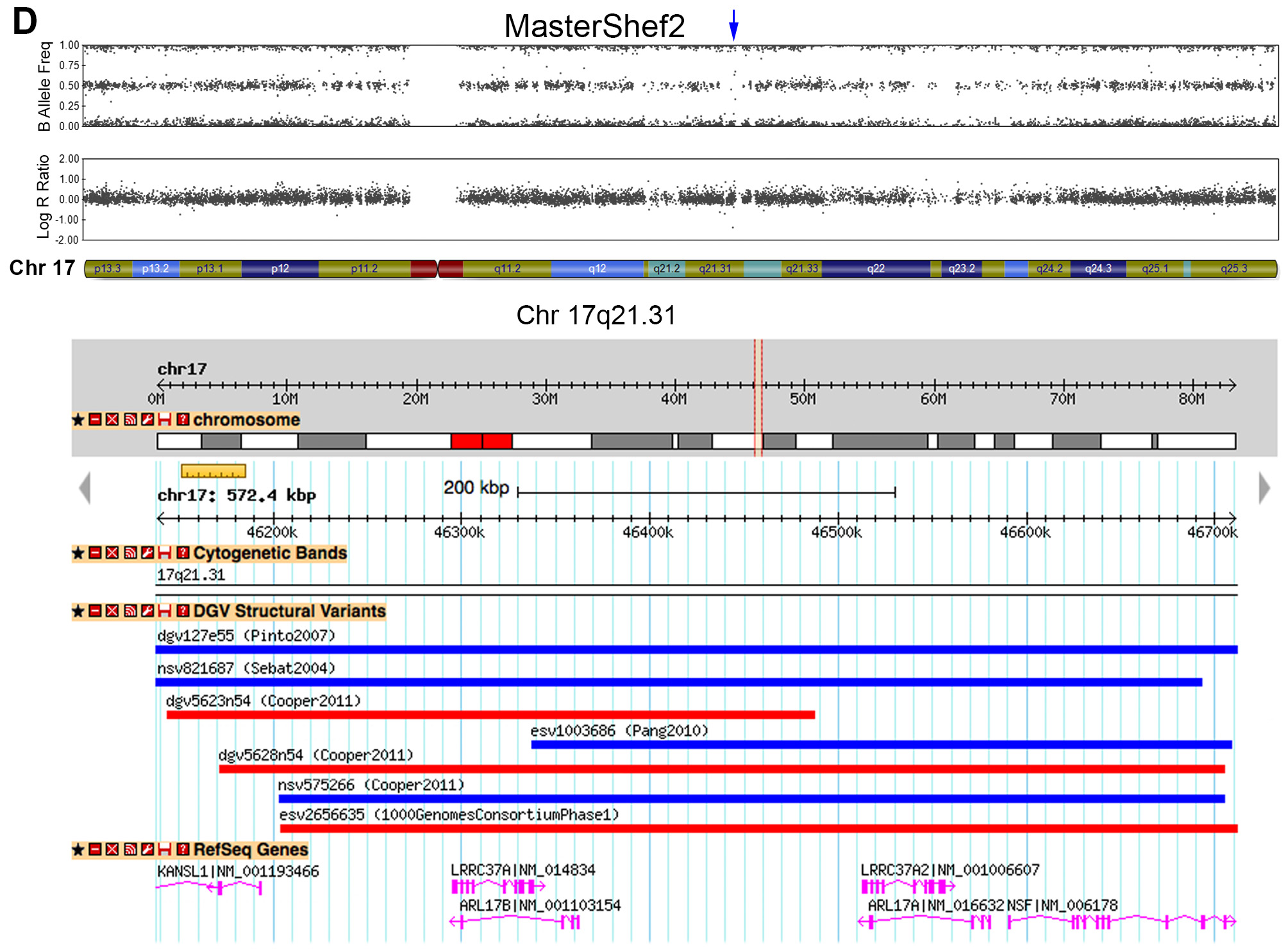


**Supplementary Figure S1: Duplications found in hESC lines that are present on the DGV.** (A) A 132.1 kb duplication on chromosome 12p11.21 was observed in two unrelated hESC lines, KCL033 and KCL040. This region does not contain any protein-coding genes, and is well represented on the DGV and relatively common (4.7%). (B) A 220.6 kb duplication was observed in Man11 hESCs spanning most of the *AKAP13* gene, but was not present in the sibling line Man12. This is a known CNV and is present on the DGV at a low frequency (0.3%). (C) A 315.4 kb duplication on chromosome 14q21.1 was observed in MasterShef7 hESCs. This CNV is not apparent in the whole chromosome 14 ideogram, but is significantly called by the KaryoStudio software. The region contains the *MDGA2* gene and is represented on the DGV by one entry. (D) A 572.4 kb duplication on chromosome 17q21.31 was observed in MasterShef2 hESCs. This CNV contained 5 coding genes, and a number of duplications and deletions have been reported on the DGV within this region and is common (9.82%).

**Supplementary Figure S2A**

**
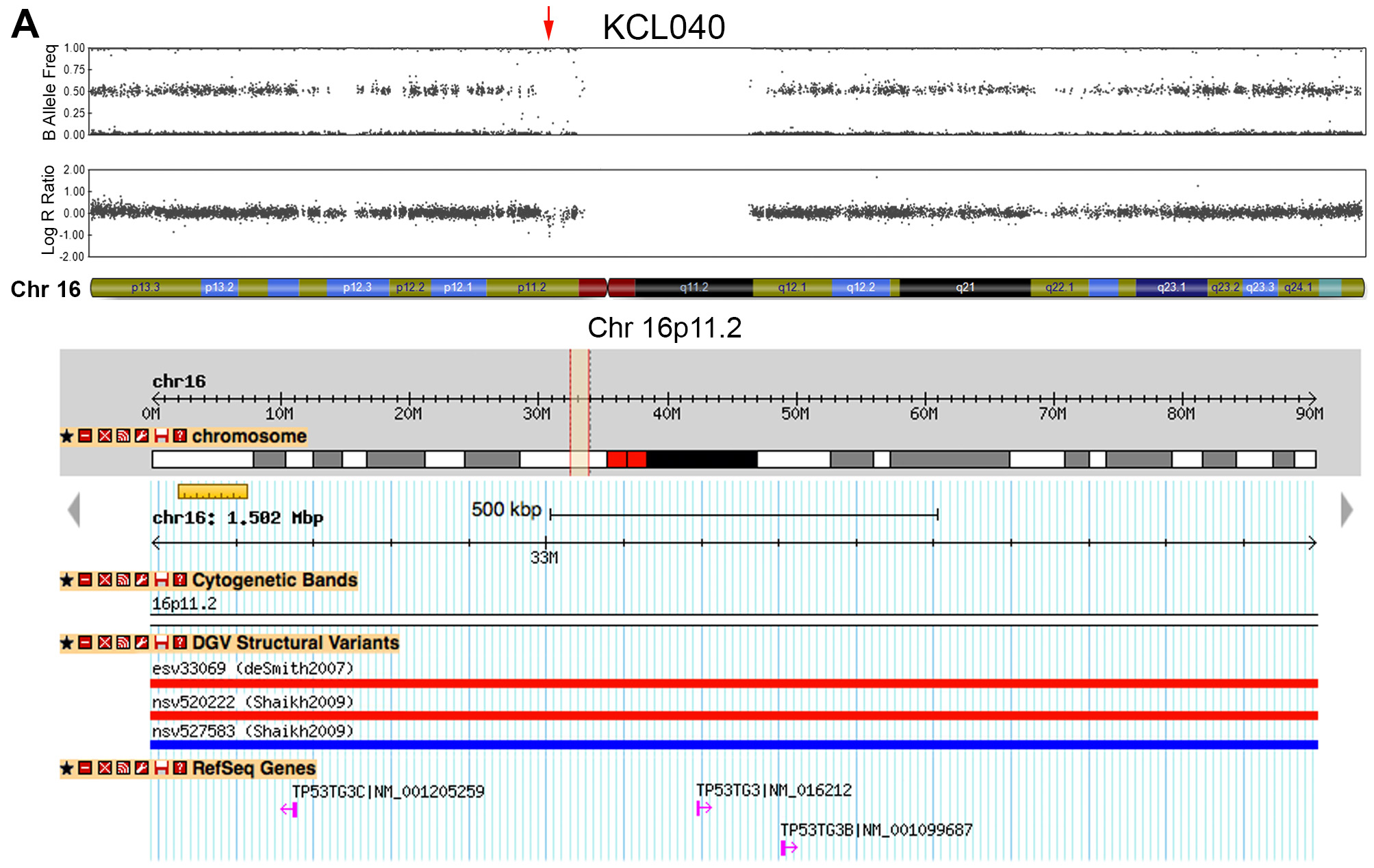
**

**Supplementary Figure S2B**

**
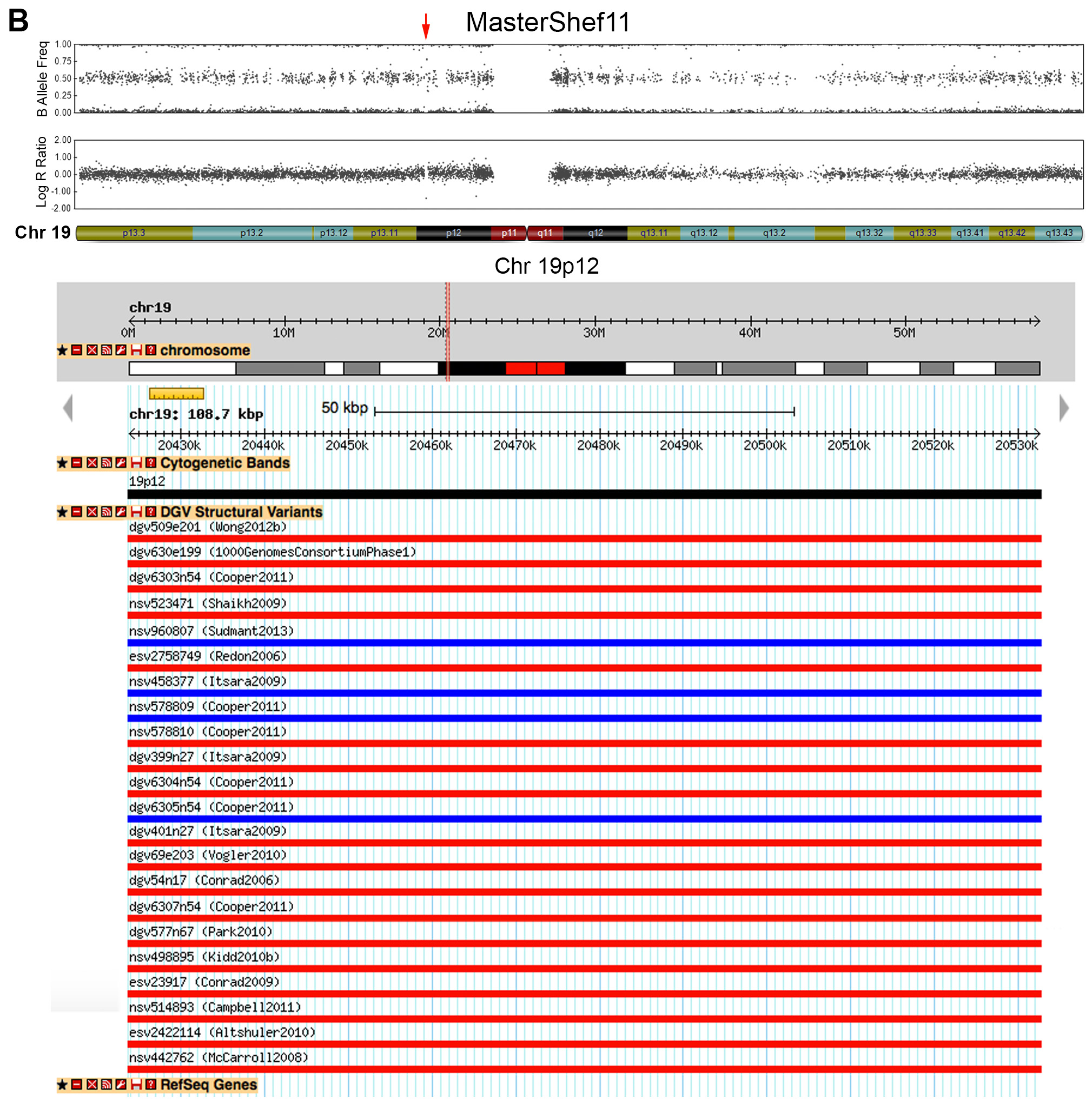
**

**Supplementary Figure S2: Deletions found in hESC lines that are present on the DGV.** (A) A 1.5 Mb deletion was observed on chromosome 16p11.2 of KCL040 hESCs containing 3 protein-coding genes. Two deletions and one duplication of this region have been reported and are present on the DGV at a relatively common frequency of 5.14%. (B) A homozygous deletion of 108.7 kb was observed on chromosome 19p12 in MasterShef11 hESCs. This region has been frequently observed to be deleted and duplicated in the human population (10.94%), and it does not contain any coding genes.

**Supplementary Figure S3A,B**

**
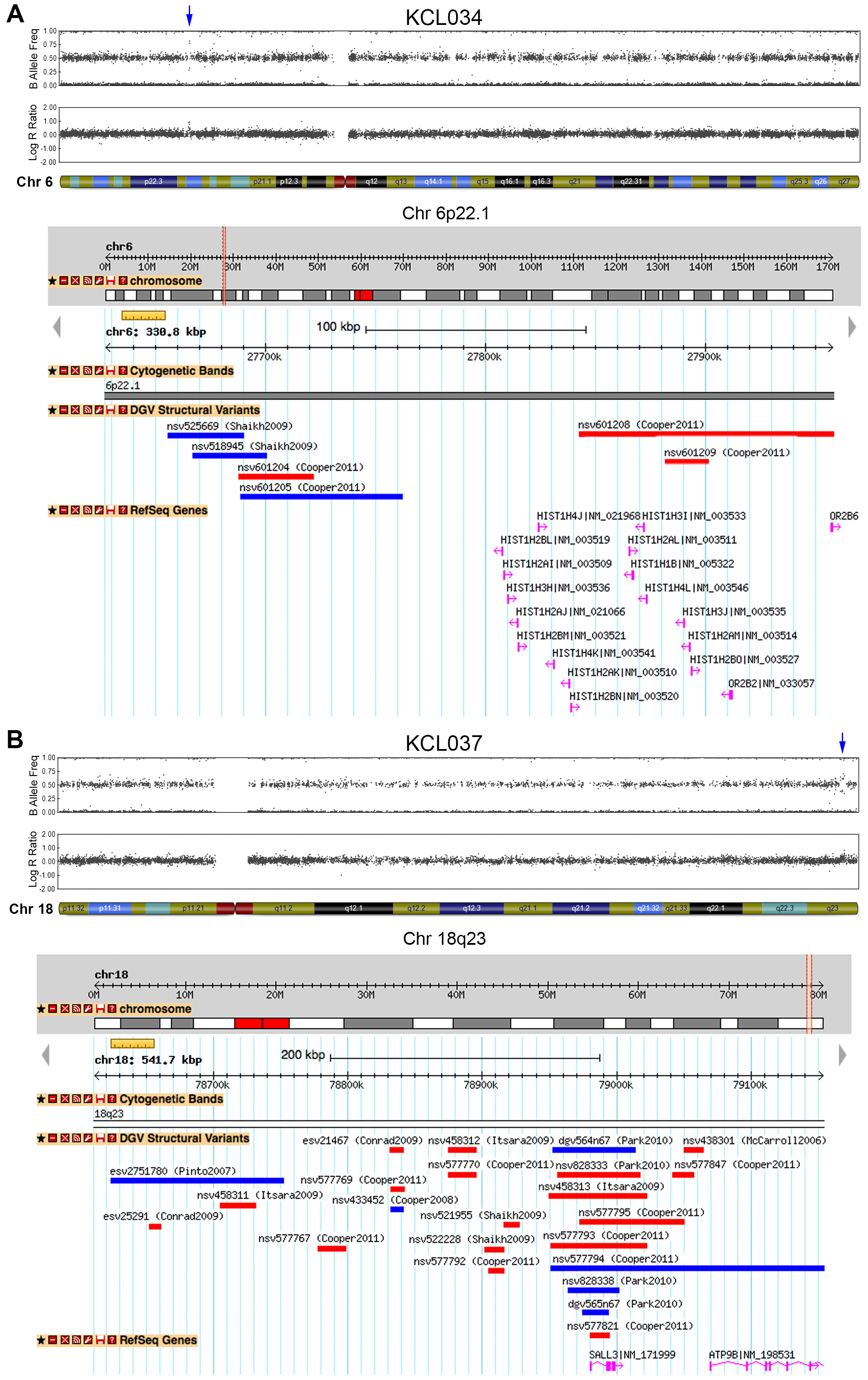
**

**Supplementary Figure S3C,D**

**
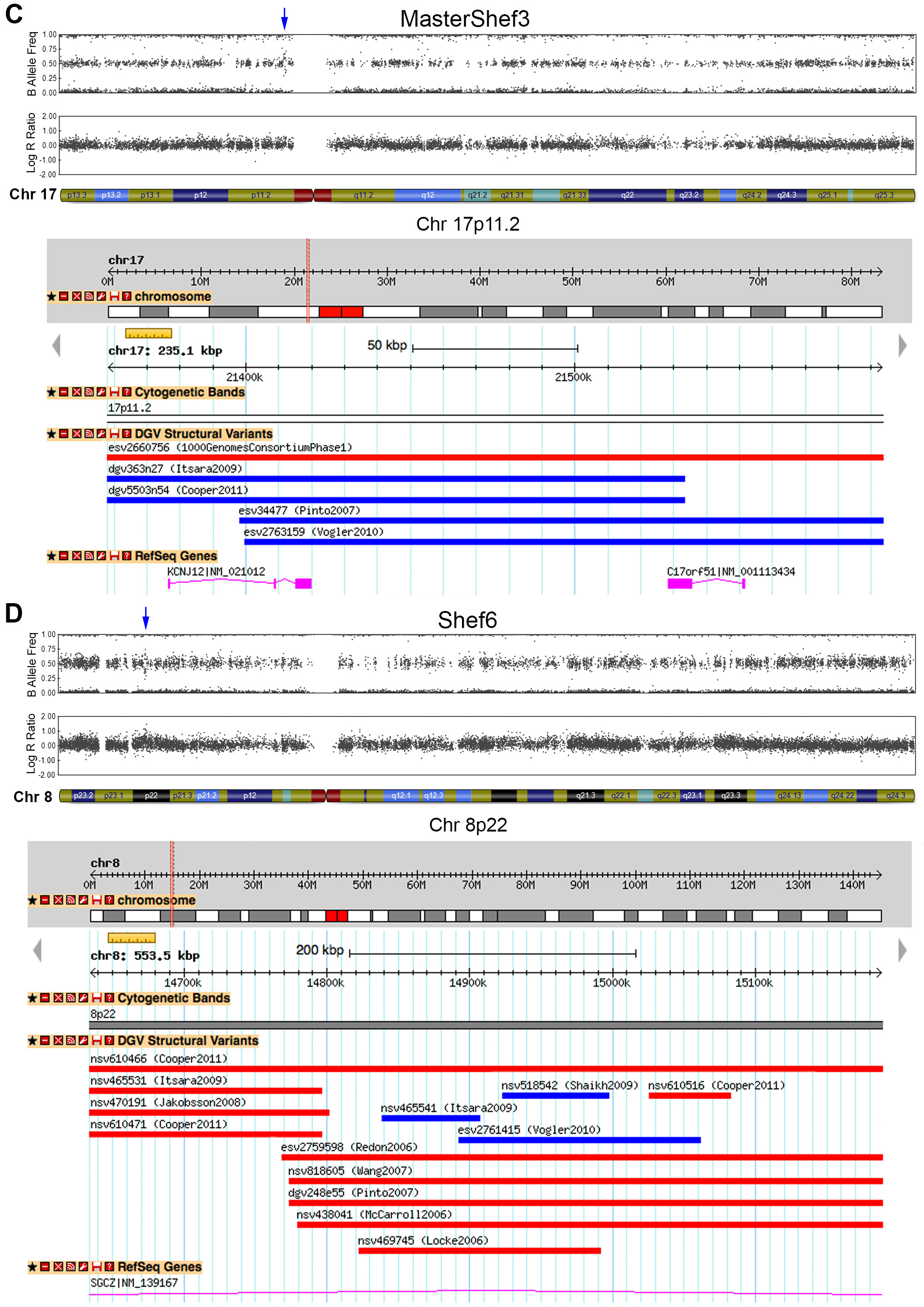
**

**Supplementary Figure S3: Duplication in hESCs that are not fully represented on the DGV.** (A) A 330.8 kb duplication on chromosome 6p22.1, containing part of the Histone 1 gene cluster, was detected in KCL034 hESCs. A duplication of this size has not been reported, but a deletion (nsv601208) spanning most of the Histone 1 cluster has been observed. (B) A 541.7 kb duplication on chromosome 18q23 was found in KCL037 hESCs. Although a duplication of this size has not been reported, a smaller duplication (nsv577794) spanning the two coding genes in the region has been observed. Only published CNVs larger that 10 kb are represented on this plot. (C) MasterShef3 harboured a 235.1 kb duplication on chromosome 17p11.2 spanning the *KCNJ12* and *C17ORF51* genes. Although a duplication of this size has not been reported, a deletion of this region is on the DGV (esv2660756), as well as several duplications covering the majority of this CNV. (D) A 553.5 kb duplication was detected within an intron of *SGCZ* on chromosome 8p22 in Shef6 hESCs. Small duplications within this CNV have been reported, and a full deletion of the region is present on the DGV (nsv610466).
